# Long‐Term Outcomes of Omniflow II and BioIntegral Xenografts for Open Aortic Reconstruction in Predominantly Infected Settings: A Multicenter Cohort Study

**DOI:** 10.1002/wjs.70559

**Published:** 2026-09-12

**Authors:** Tijmen W. Kraai, Eline I. Reinders Folmer, Barzi Gareb, David J. Liesker, Ben R. Saleem, Clark J. Zeebregts, Vascular Xenograft Research Group

**Affiliations:** ^1^ Department of Surgery (Division of Vascular Surgery) University Medical Center Groningen University of Groningen Groningen the Netherlands; ^2^ Department of Oral‐ and Maxillofacial Surgery University Medical Center Groningen University of Groningen Groningen the Netherlands; ^3^ Departments of Surgery (Division of Vascular Surgery) St Antonius Hospital Utrecht the Netherlands

**Keywords:** aortic reconstruction, biointegral, omniflow II, vascular graft infection, xenograft

## Abstract

**Objective:**

Evidence surrounding conduit selection for open in situ aortic reconstruction remains limited particularly for xenograft use in infected settings. We evaluated long‐term outcomes after open aortic and aorto‐iliac reconstruction using BioIntegral No‐React bovine grafts and Omniflow II biosynthetic grafts in patients unsuitable for autologous vein reconstruction.

**Methods:**

This multicenter cohort study combined a retrospective Omniflow II cohort from six Dutch hospitals with a prospectively collected BioIntegral registry. Patients undergoing open aortic, aortoiliac, or aortofemoral reconstruction in infected and selected noninfected settings between 2014 and 2021 were included; peripheral bypasses and arteriovenous access procedures were excluded. The primary outcome was graft infection. Secondary outcomes included patency rates (primary, primary‐assisted, and secondary), mortality, freedom from major amputation, and 30‐day adverse events. Time‐to‐event outcomes were analyzed using Kaplan–Meier methods and univariate Cox regression.

**Results:**

Forty‐four patients were included (BioIntegral *n* = 21, Omniflow II *n* = 23). Baseline characteristics were comparable except for a lower body mass index in the Omniflow group (26.8 vs. 23.9 kg/m^2^; *p* = 0.046). Thirty‐day mortality was 19.0% for BioIntegral and 30.4% for Omniflow II. Infection from infection at 12, 24, and 60 months was 88.2%, 63.7%, and 43.7% for BioIntegral versus 87.1%, 79.1%, and 79.1% for Omniflow II (log‐rank *p* = 0.13). Primary patency at 60 months was 49.9% versus 55.5% (*p* = 0.48) and secondary patency was 85.7% versus 72.4% (*p* = 0.24). Overall survival at 60 months was 25.0% versus 47.3% (*p* = 0.41). Freedom from major amputation at 60 months was 81.8% versus 88.8% (*p* = 0.59). Graft type was not associated with significant differences in univariate analyses.

**Conclusions:**

In open aortic reconstruction performed predominantly for vascular graft or endograft infection, BioIntegral and Omniflow II xenografts showed comparable long‐term outcomes without statistically significant differences in patency, mortality or freedom from major amputation. Findings should be interpreted as descriptive given the small sample size and mixed indications. Late graft infections highlight the need for prolonged surveillance after reconstruction.

## 1 Introduction

The incidence of open aortic surgery has declined substantially following the widespread adoption of endovascular aneurysm repair (EVAR) [1]. Nonetheless, current guidelines continue to recommend open aortic reconstruction for selected indications, including unfavorable anatomy for EVAR, infected aortic grafts and infective native aortic aneurysms (INAA) [2, 3].

Management of infected aortic grafts and aneurysms remains challenging, as the optimal conduit for reconstruction is still debated. Synthetic graft materials have been associated with reinfection rates of up to 29%, contributing substantially to postoperative morbidity and mortality [4, 5, 6]. Autologous vein reconstruction, often in the form of the neoaortoiliac system (NAIS) procedure, is associated with markedly lower reinfection rates of approximately 5%, but its use is limited by vein availability, harvest‐site morbidity, and increased operative complexity and duration [6, 7, 8]. Consequently, there is a clear clinical need for readily available, infection‐resistant materials.

Several off‐the‐shelf alternatives have been introduced, including different xenografts. Among these, the Omniflow II biosynthetic graft (LeMaitre Vascular Inc., Burlington, MA, USA) has demonstrated promising outcomes in peripheral settings [9, 10]. A recent meta‐analysis by El‐Diaz et al. reported a pooled freedom from reinfection of 97.7% following aortic reconstruction with Omniflow II [11]. However, the available evidence remains limited, as this analysis included only 60 patients and lacked comparative long‐term data.

Another xenograft option, the BioIntegral No‐React bovine pericardial graft (BioIntegral Surgical Inc., Mississauga, Canada), has shown favorable short‐term infection‐related outcomes in aortic reconstruction. Nevertheless, questions regarding long‐term durability and late infectious complications persist, as current existing studies are primarily small series with limited follow‐up [12, 13]. Although Reinders Folmer et al. reported acceptable 1‐year outcomes, the absence of long‐term data warrants further investigation [14].

Given the paucity of comparative long‐term data on these xenografts, the primary objective of this study was to evaluate long‐term outcomes for the BioIntegral and Omniflow II grafts in aortic reconstruction in several Dutch vascular centers. Secondary objectives included assessing potential differences in clinical performance between both grafts. We hypothesized that both conduits would demonstrate comparable long‐term outcomes.

## 2 Methods

### 2.1 Study Design and Population

This study comprised a multicenter cohort of patients undergoing open aortic reconstruction using xenograft conduits. A retrospective cohort of patients treated with Omniflow II biosynthetic grafts in six Dutch hospitals was combined with a prospectively collected cohort of patients treated with BioIntegral bovine pericardial grafts in seven Dutch hospitals. Although seven centers participated in the prospective BioIntegral registry, two centers delivered prolonged follow‐up for the present analysis. Patients undergoing aortic, aortoiliac, or aortofemoral reconstruction between 2014 and 2021 in infected and noninfected settings, where conventional reconstruction options were considered unsuitable, were eligible for inclusion. Patients were deemed unsuitable for autologous vein reconstruction (including the NAIS procedure) based on operative risk, venous anatomy, extent of reconstruction, and anticipated morbidity of vein harvest. Final conduit selection was not protocolized, reflecting real‐world individualized surgeon‐ and center‐level decision‐making. Patients undergoing peripheral bypass procedures or arteriovenous access surgery were excluded.

The study was approved by all institutional review boards of participating centers (METC registration numbers: 2017/830 and 2021/494) and conducted in accordance with the Declaration of Helsinki. As the study did not fall under the Dutch Medical Research Involving Human Subjects Act (WMO), additional informed consent was not required. This study was performed in accordance with STROBE guidelines [15].

### 2.2 Data Collection

Patient‐level data were collected from electronic medical records and included demographics, cardiovascular comorbidities, clinical presentation, infection status, number of prior aortic operations, procedural urgency (acute or elective), anatomical level of reconstruction, antimicrobial therapy, anticoagulant and antiplatelet use, and preoperative laboratory values. Variables were analyzed and reported by graft type.

### 2.3 Outcome Measures

The primary outcome was graft‐related infection. Secondary outcomes included patency (primary, primary‐assisted and secondary), (graft‐related) mortality, and major amputation (above‐ankle). 30‐day adverse events were recorded, including mortality, bleeding at surgical site, cardiac adverse events, graft occlusion, wound infection, and cerebrovascular events (cerebrovascular accident or transient ischemic attack).

Graft infection was defined according to the Management of Aortic Graft Infection Collaboration (MAGIC) criteria and was based on microbiological evidence, clinical signs and/or imaging findings indicative of infection [16]. Patient follow‐up was standardized at 3 and 12 months postoperatively, including physical examination and routine duplex ultrasound. Thereafter, annual follow‐up was performed for as long as clinically indicated. Computed tomography angiography and/or nuclear imaging was performed when indicated particularly in case of suspected graft infection or graft‐related complications.

### 2.4 Device Characteristics

The bovine pericardial graft was the No‐React nonvalved conduit (BioIntegral Surgical Inc.), a fully biologic stentless xenograft composed of glutaraldehyde‐treated bovine pericardium. Its surface undergoes treatment with heparin rinsing and glutaraldehyde fixation to improve biocompatibility and reduce antigenicity. Bifurcations with diameters of 20/10 mm or 18/9 mm were used.

The Omniflow II biosynthetic graft (LeMaitre Vascular Inc.) is a composite xenograft consisting of a polyester mesh endoskeleton surrounded by ovine fibro‐collagenous tissue. The graft is formed by subcutaneous implantation of the mesh in sheep after which the collagenous tissue is explanted and treated with glutaraldehyde to crosslink collagen fibers and reduce antigenicity. Two Omniflow 8‐mm grafts were surgically anastomosed proximally to create a common trunk suitable for aortic inflow after which the distal limbs were anastomosed to the peripheral arteries (Figure 1). Graft selection and configuration were determined by the operating surgeon, center‐specific availability, and practice.

**FIGURE 1:**
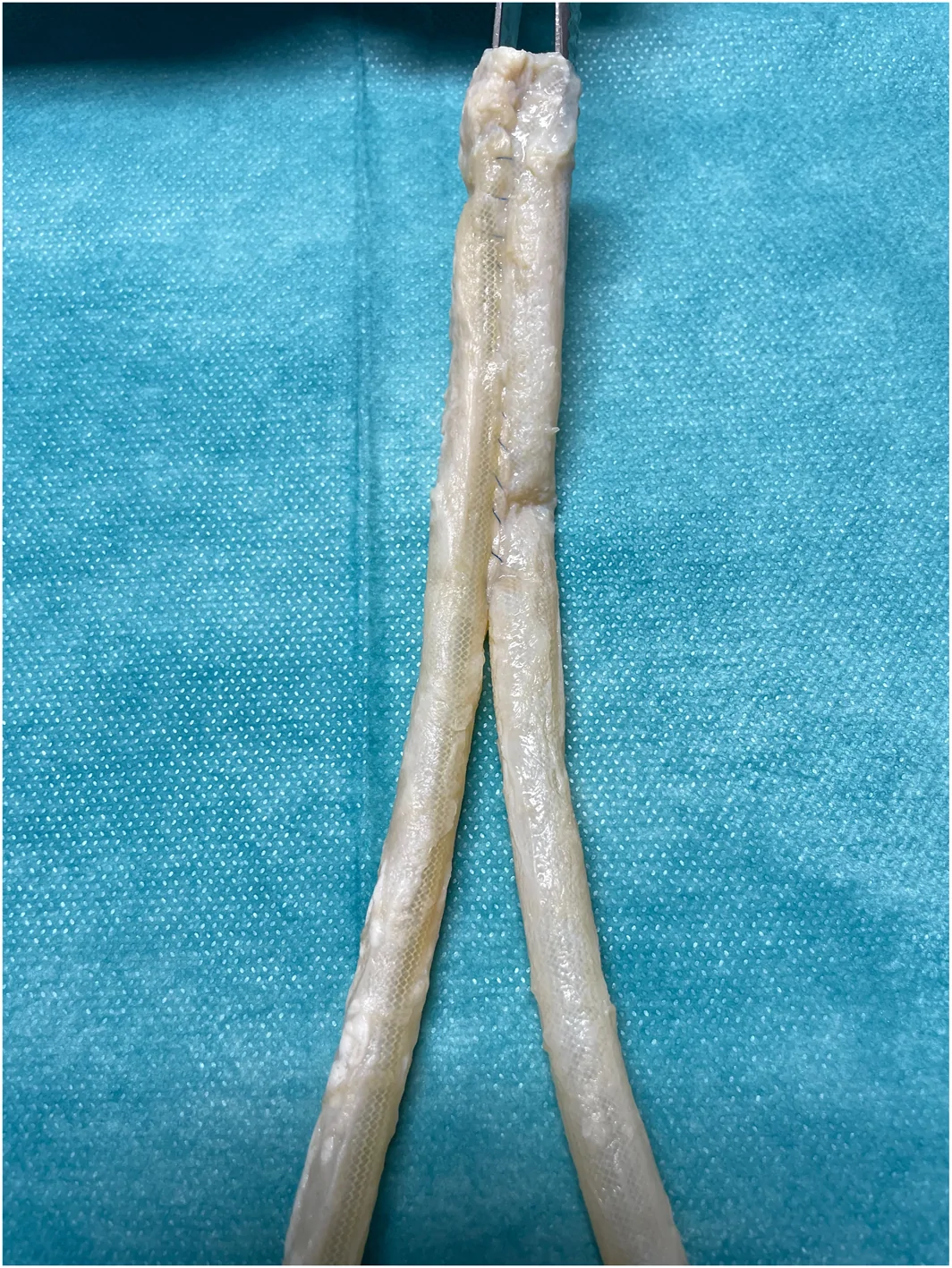
Example of how an Omniflow bifurcation graft is created from two 8‐mm grafts. The two limbs were anastomosed proximally to create a common trunk suitable for aortic inflow, and the distal limbs were anastomosed to the iliac or femoral arteries.

Postoperative antithrombotic therapy was not conduit‐specific but was prescribed according to local practice, standard cardiovascular secondary prevention, and individual indications for anticoagulation.

### 2.5 Statistical Analysis

Categorical variables are presented as frequencies with percentages and were compared using Fisher's exact test. Continuous variables are presented as means with standard deviations when normally distributed and were compared using the independent‐samples *t*‐test. Skewed data are presented as medians with interquartile ranges and were compared using the Mann–Whitney *U* test. Data distributions were assessed visually using histograms and Q‐Q‐plots and formally tested using the Shapiro–Wilk test.

Time‐to‐event outcomes were analyzed using Kaplan–Meier survival analysis with curves stratified by graft type. Differences between groups were assessed using the log‐rank test. Cox proportional hazards regression models were used to estimate the unadjusted association between graft type and each outcome. The proportional hazard assumption was evaluated using Schoenfeld residuals. Patients were censored at the time of last follow‐up or death. A two‐sided *p* value < 0.05 was considered statistically significant for all analyses.

All statistical analyses were performed using R version 4.3.5 (R Foundation for Statistical Computing, Vienna, Austria) with the *tableone‐, survival‐, survminer‐, CompareGroups‐,* and *ggplot2‐*packages.

## 3 Results

### 3.1 Baseline Patient‐ and Intraoperative Characteristics

All 44 eligible patients were included, of whom 21 underwent reconstruction with a BioIntegral graft and 23 with an Omniflow graft, with a mean follow‐up of 29 months (range 0–99) and 0 months of follow‐up representing early postoperative deaths. No patients were lost to follow‐up. Baseline characteristics were largely comparable between groups. Body mass index was lower in the Omniflow group (26.8 vs. 23.9 kg/m^2^; *p* = 0.046), whereas no other statistically significant differences were observed between other demographics and patient characteristics (Table 1).

**TABLE 1:** Baseline Characteristics by used material.

	BioIntegral	Omniflow	*p*‐value
*n* (%)	*N =* 21	*N =* 23	
Age [median, IQR's]	70.0 [61.0; 73.0]	72.0 [64.0; 73.0]	0.750
Male sex	18 (85.7%)	16 (69.6%)	0.287
BMI (mean, SD)	26.8 (5.19)	23.9 (3.58)	**0.046**
Smoking status:			0.371
Current, < 1 pack a day	7 (33.3%)	10 (45.5%)	
Current, > 1 pack a day	3 (14.3%)	0 (0.00%)	
Quit 1–10 years ago	5 (23.8%)	6 (27.3%)	
Never or quit > 10 years ago	6 (28.6%)	6 (27.3%)	
Hypertension status:			0.162
> 2 antihypertensive drugs, or uncontrolled	1 (4.76%)	6 (26.1%)	
2 antihypertensive drugs	3 (14.3%)	5 (21.7%)	
1 antihypertensive drug	12 (57.1%)	7 (30.4%)	
None	5 (23.8%)	5 (21.7%)	
Hypertension	16 (76.2%)	18 (78.3%)	1.000
Diabetes mellitus	8 (38.1%)	6 (26.1%)	0.596
Hyperlipidemia	13 (61.9%)	18 (78.3%)	0.391
CVA or TIA in history	3 (14.3%)	6 (26.1%)	0.462
ASA‐score:			0.056
2	0 (0.00%)	5 (21.7%)	
3	13 (68.4%)	10 (43.5%)	
4	6 (31.6%)	6 (26.1%)	
5	0 (0.00%)	2 (8.70%)	
Cardiac disease	12 (57.1%)	12 (52.2%)	0.978
Anticoagulation usage	5 (26.3%)	8 (34.8%)	0.798
Antiplatelet usage	11 (57.9%)	17 (73.9%)	0.443
Number of prior aortic surgeries	1.33 (1.28)	1.52 (1.86)	0.695
Follow‐up [median, IQR's]	18.0 [7.00; 52.0]	23.0 [0.00; 57.5]	0.897

*Note:* Bold *p*‐values are statistically significant.

Abbreviations: ASA, American Society of Anesthesiologists; BMI, Body Mass Index; CVA, Cerebrovascular Accident; IQR, Interquartile Ranges (25th percentile, 75th percentile); SD, Standard Deviation; TIA, Transient Ischemic Attack.

The level of aortic reconstruction did not differ significantly between groups (*p* = 0.10), with aortobifemoral and aortobiiliac reconstructions performed most frequently. Surgical indications were similar between groups (*p* = 0.13) with vascular graft infection being the predominant indication. The remaining procedures consisted of primary reconstructions for infective native aneurysms and selected reconstructions performed outside an overtly infected field, when standard reconstruction options were considered unsuitable or less desirable by the local treatment team. The proportion of acute versus elective procedures was also comparable (*p* = 0.83). Preoperative laboratory results did not differ significantly between groups (all *p* > 0.05). In addition, prior implanted graft material, operative time, and intraoperative blood loss were similar between groups (Table 2).

**TABLE 2:** Intraoperative characteristics by used material.

	BioIntegral	Omniflow	p.overall
	*N* = 21	*N* = 23	
Location:			0.099
Aorta only	1 (4.76%)	0 (0.00%)	
Aorta bifemoral	8 (38.1%)	15 (65.2%)	
Aorta bi‐iliac	12 (57.1%)	8 (34.8%)	
Indication:			0.128
VGEI	18 (85.7%)	14 (60.9%)	
Infective native aortic aneurysm	3 (14.3%)	4 (17.4%)	
Noninfective native aortic aneurysm	0 (0.00%)	4 (13.0%)	
Urgency of surgery:			0.828
Acute	8 (38.1%)	7 (30.4%)	
Elective	13 (61.9%)	16 (69.6%)	
Leukocytes	10.8 [9.00; 13.5]	9.00 [7.30; 12.4]	0.333
CRP (mg/L)	50.0 [23.0; 166]	81.5 [26.5; 130]	0.560
Hb (mmol/L)	6.80 (1.10)	6.72 (1.13)	0.822
eGFR (ml/min/1,73 m^2^)	72.3 (24.0)	70.2 (21.6)	0.771
Previous in situ material:			0.196
Dacron	11 (52.4%)	6 (26.1%)	
EVAR	6 (28.6%)	6 (26.1%)	
Bovine	0 (0.00%)	1 (4.35%)	
None	3 (14.3%)	9 (39.1%)	
Silver coated dacron	1 (4.76%)	1 (4.35%)	
Surgery time (min)	290 [240; 386]	316 [268; 472]	0.350
Blood loss (mL)	2900 [1600; 4300]	3000 [1300; 3800]	0.722

Abbreviations: CRP, C‐Reactive Protein; eGFR, estimated Glomerular Filtration Rate; EVAR, Endovascular Aneurysm Repair; Hb, Hemoglobin; VGEI, Vascular Graft and/or Endograft Infection.

### 3.2 Short‐Term Outcomes (< 30 days)

Thirty‐day mortality occurred in 19.0% (4/21) of patients in the BioIntegral group and 30.4% (7/23) in the Omniflow group (*p* = 0.60). In the BioIntegral group, two deaths were considered graft‐related: One due to an iliac artery occlusion which was successfully treated with an embolectomy. After subsequent critical limb ischemia on postoperative day 4, no further treatment options were considered in accordance with the patient. The other patient died due to anastomotic bleeding at the surgical site. In the Omniflow group, three deaths were classified as graft‐related, including one associated with progression of peripheral arterial disease. Following multidisciplinary discussion and in accordance with the patient's wishes, no further revascularization was undertaken. Two patients died due to bleeding complications in the setting of an aortoduodenal fistula.

Deaths not classified as graft‐related were attributed to gastrointestinal complications, a cardiac event, and ongoing sepsis without established graft involvement. Two patients died following gastrointestinal complications: One after extensive low‐flow bowel ischemia due to major operative blood loss and hemodynamic instability, and one after a prolonged ICU course complicated by suspected ischemic colitis, which led to abdominal sepsis, renal failure, and ultimately multiple organ failure following aortoenteric fistula repair. The patient that remained septic after the initial procedure underwent secondary drainage of a residual fluid collection, which showed no signs of active bleeding or clear ongoing graft infection. The patient subsequently developed a fatal cardiac event in the setting of ongoing sepsis. No autopsy was performed; therefore, a delayed graft‐related complication could neither be confirmed nor fully excluded.

Other adverse events included in‐hospital cardiac arrests in one patient in each group. Delirium occurred in one patient in the Omniflow group and in none of the BioIntegral group. Graft occlusion occurred in two patients in the Omniflow group and in one patient in the BioIntegral group. One Omniflow graft occlusion was successfully treated with redo surgery, whereas the remaining two occlusions resulted in death. No cerebrovascular adverse events, urinary tract infections or wound infections occurred within 30 days in either group. None of the short‐term outcomes differed significantly between groups (Table 3).

**TABLE 3:** Short‐term (< 30‐day) outcomes by used material.

	BioIntegral	Omniflow	*p*‐value
	*n =* 21	*n =* 23	
Mortality	4 (19.0%)	7 (30.4%)	0.601
CVA or TIA	—	—	.
Cardiac adverse events	1 (4.76%)	1 (4.35%)	1.000
Bleeding at surgery site	3 (14.3%)	2 (8.70%)	0.658
Delirium	0 (0.00%)	1 (4.35%)	1.000
Urinary tract infection	—	—	.
Wound infection	—	—	.
Occlusion	1 (4.76%)	2 (8.70%)	1.000

Abbreviations: CVA, Cerebrovascular Accident; TIA, Transient Ischemic Attack.

### 3.3 Long‐Term Outcomes

#### 3.3.1 Infection

Preoperative infection characteristics at patient‐level are portrayed in Supporting Information S2: Tables 2. Infection events, including both primary and recurrent graft infections, were analyzed in the overall cohort (*n* = 44) with a total of 10 events observed during follow‐up. In the BioIntegral group, freedom from infection was 88.2%, 63.7%, and 43.7% at 12, 24, and 60 months (numbers at risk: 13, 7, and 4, respectively), whereas corresponding estimates in the Omniflow group were 87.1%, 79.1%, and 79.1% (numbers at risk: 13, 9, and 4, respectively). Kaplan–Meier curves did not differ significantly between groups (log‐rank *p* = 0.13) (Figure 2). In univariate Cox proportional hazards analysis, graft type was not significantly associated with graft infection (HR0.37, 95%CI 0.09–1.43; *p* = 0.15).

**FIGURE 2:**
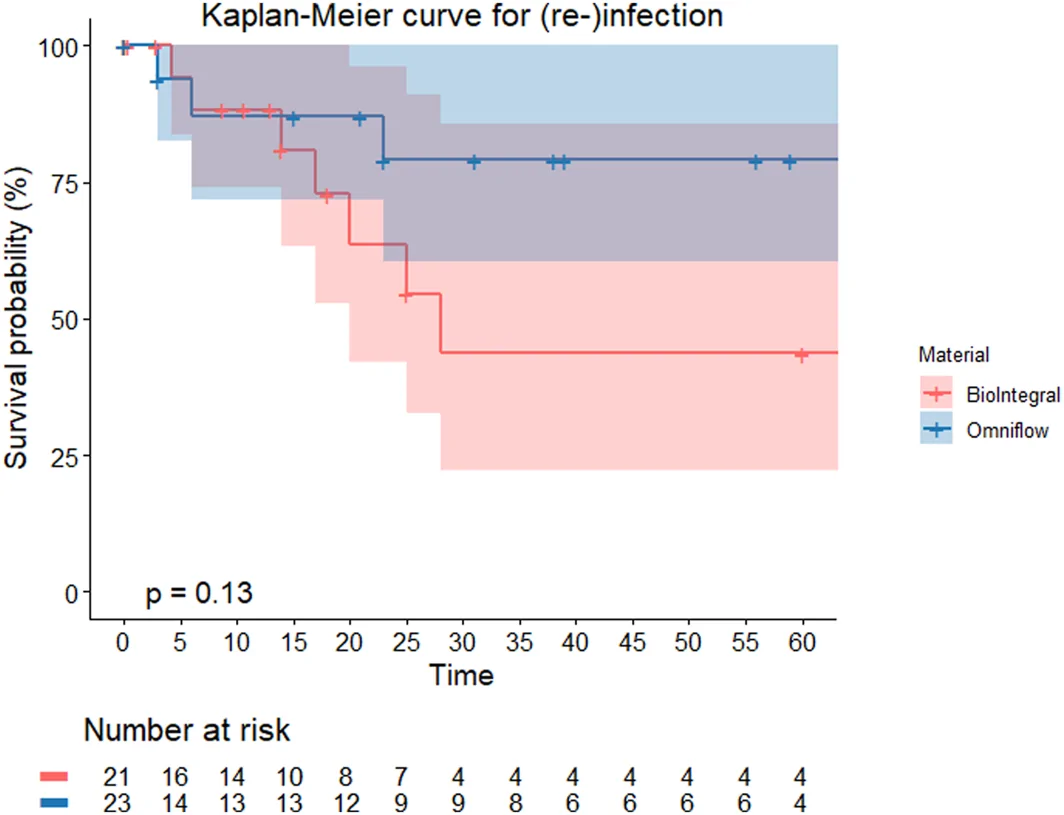
Kaplan–Meier curves of (re‐)infection rates for Omniflow and BioIntegral. Time expressed in months. Subgroups were compared using the log‐rank test, with corresponding *p*‐values mentioned in the figure.

All graft infections were treated with prolonged culture‐directed antibiotic therapy. Ultimately, two BioIntegral grafts were explanted because of persistent or recurrent infection despite antibiotic treatment. Two patients who received BioIntegral grafts died due to persistent infection and abstinence of further treatment. The remaining infection events were managed without graft removal using antibiotics with or without additional surgical drainage or debridement when indicated.

#### 3.3.2 Patency

Primary patency was assessed in the overall cohort (*n* = 44) with 14 loss‐of‐primary‐patency events observed during follow‐up. In the BioIntegral group, primary patency was 85.4%, 77.7%, and 49.9% at 12, 24, and 60 months (numbers at risk: 13, 8, and 4, respectively), whereas corresponding estimates in the Omniflow group were 62.4%, 55.5%, and 55.5% (numbers at risk: 10, 7, 3, respectively). Kaplan–Meier curves did not differ significantly between groups (log‐rank *p* = 0.48) (Figure 3a). In univariate Cox proportional hazards analysis, graft type was not significantly associated with loss of primary patency (HR1.46, 95%CI 0.51–4.23; *p* = 0.48).

**FIGURE 3:**
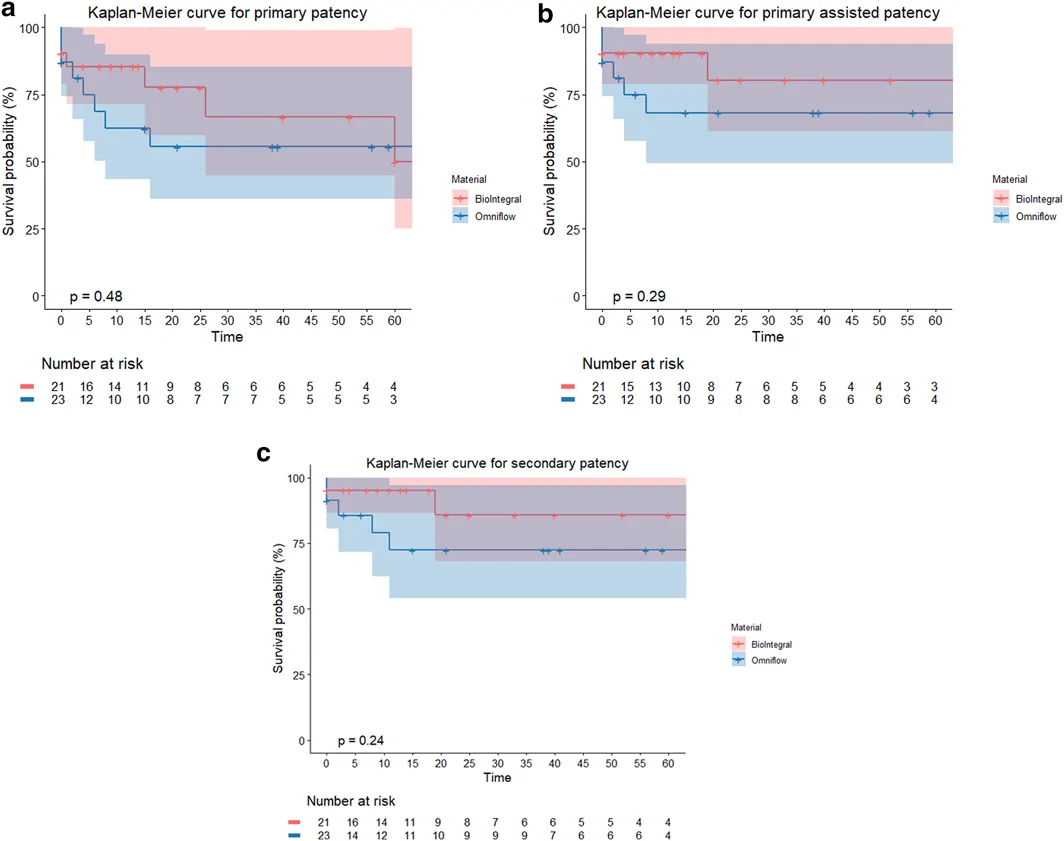
abc—Kaplan–Meier curves of patency rates (a; primary patency, b; primary assisted patency, c; secondary patency). Time is expressed in months. Subgroups were compared using the log‐rank test, with corresponding *p*‐values mentioned in the figures.

Primary assisted patency was assessed in the overall cohort (*n* = 44) with nine events observed during follow‐up. In the BioIntegral group, primary assisted patency was 90.5%, 80.4%, and 80.4% at 12, 24 and 60 months (numbers at risk: 12, 7, and 3, respectively), whereas corresponding estimates in the Omniflow group were 68.1%, 68.1%, and 68.1% (numbers at risk: 10, 8, and 4, respectively). Kaplan–Meier curves did not differ significantly between groups (log‐rank *p* = 0.29) (Figure 3b). In univariate Cox proportional hazards analysis, graft type was not significantly associated with loss of primary assisted patency (HR2.05, 95%CI 0.51–8.21; *p* = 0.31). Secondary patency was assessed in the overall cohort (*n* = 44) with seven events observed during follow‐up. In the BioIntegral group, secondary patency was 95.2%, 85.7%, and 85.7% at 12, 24, and 60 months (numbers at risk: 13, 8, and 4, respectively), whereas corresponding estimates in the Omniflow group were 72.4%, 72.4%, and 72.4% (numbers at risk: 11, 9, and 4, respectively). Kaplan–Meier curves did not differ significantly between groups (log‐rank *p* = 0.24) (Figure 3c). In univariate Cox proportional hazards analysis, graft type was not significantly associated with loss of secondary patency (HR2.57, 95%CI 0.50–13.28; *p* = 0.26).

Loss of primary patency was mainly attributable to graft occlusion, which occurred in nine cases (5 Omniflow and 4 BioIntegral), all within 9 months postoperatively. Occlusions were managed by thrombectomy, thrombolysis, thromboendarterectomy, embolectomy, or bypass revision, although two BioIntegral occlusions ultimately led to major amputation. Additional causes of primary patency loss included late graft stenosis, all occurring after 12 months and treated by thromboendarterectomy. Loss of secondary patency resulted from graft abandonment after failure leading to major amputation, or explantation for persistent graft infection.

#### 3.3.3 Mortality

Overall survival was assessed in the total cohort (*n* = 44) with 25 deaths observed during follow‐up. In the BioIntegral group, overall survival was 66.7%, 50.0%, and 25.0% at 12, 24, and 60 months (numbers at risk: 13, 9, and 4, respectively), whereas corresponding estimates in the Omniflow group were 60.9%, 60.9%, and 47.3% (numbers at risk: 14, 11, and 5, respectively). Kaplan–Meier survival curves did not differ significantly between groups (log‐rank *p* = 0.41) (Figure 4a). In univariate Cox proportional hazards analysis, graft type was not significantly associated with all‐cause mortality (HR0.76, 95%CI 0.34–1.67; *p* = 0.49).

**FIGURE 4:**
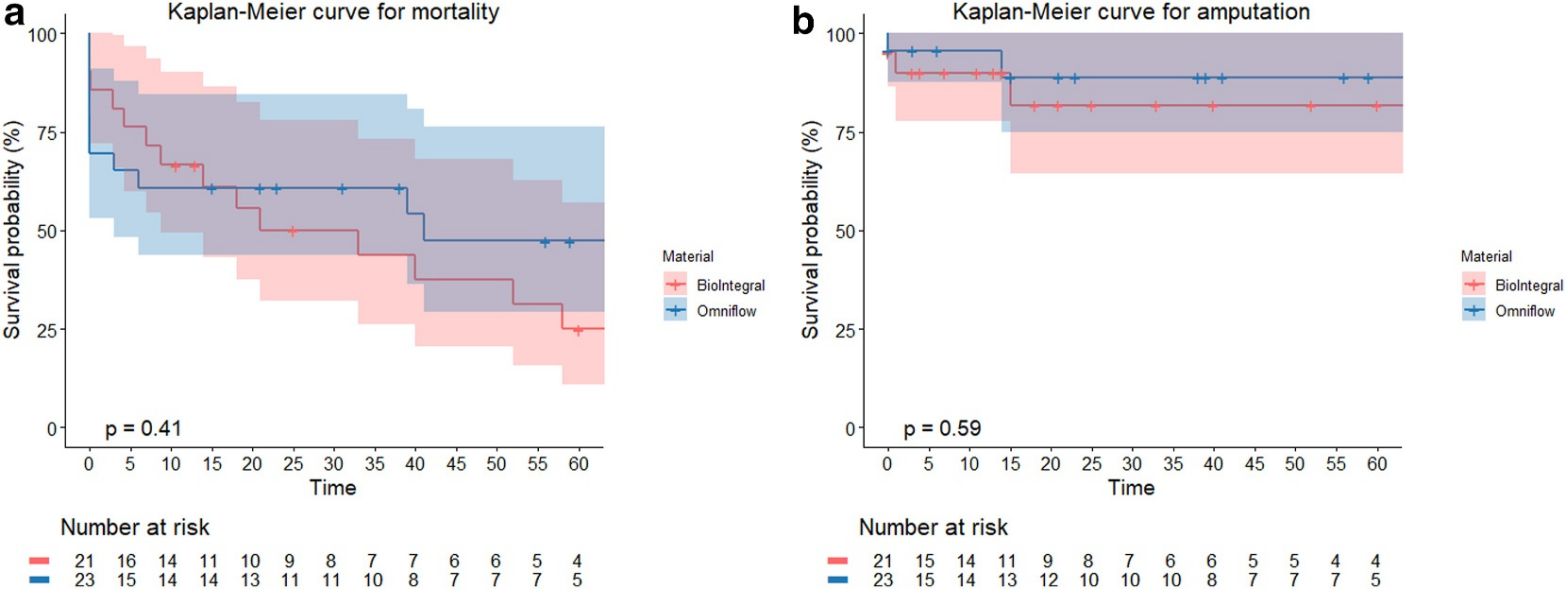
ab—Kaplan–Meier curves of mortality (a) and major amputation (b) for Omniflow and BioIntegral. Time is expressed in months. Subgroups were compared using the log‐rank test, with corresponding *p*‐value mentioned in the figures.

#### 3.3.4 Amputation

Freedom from major amputation was assessed in the overall cohort (*n* = 44) with five events observed during follow‐up. In the BioIntegral group, freedom from major amputation was 89.9%, 81.8%, and 81.8% at 12, 24, and 60 months (numbers at risk: 13, 8, and 4, respectively), whereas corresponding estimates in the Omniflow group were 95.7%, 88.8%, and 88.8% (numbers at risk: 14, 10, and 5, respectively). Kaplan–Meier curves did not differ significantly between groups (log‐rank *p* = 0.59) (Figure 4b). In univariate Cox proportional hazards analysis, graft type was not significantly associated with major amputation (HR0.61; 95%CI 0.10–3.70; *p* = 0.60).

Exact Kaplan–Meier estimates with corresponding 95% confidence intervals at 12, 24, and 60 months and corresponding numbers at risk at each time interval, for both the entire cohort as well as per subgroup, are presented in Supporting Information S1: Table 1 for each long‐term outcome.

## 4 Discussion

In this multicenter cohort study of patients undergoing aortic reconstruction with xenograft conduits, BioIntegral and Omniflow II demonstrated comparable clinical outcomes. No statistically significant differences were observed between graft types with respect to short‐term and long‐term outcomes. This study provides clinically relevant long‐term outcome data in a rare and high‐risk surgical setting where evidence remains sparse.

The use of xenograft conduits for aortic reconstruction is currently underrepresented in the literature [11]. Evidence regarding outcomes of bovine pericardial grafts for aortic reconstruction is similarly limited, as existing studies primarily consist of small cohorts with short follow‐up [17, 18, 19]. By directly comparing these two xenograft conduits with extended follow‐up, the present study contributes additional data to an evolving but still limited evidence base.

Infection remains one of the most feared complications following aortic reconstruction and is a major contributor to postoperative morbidity and mortality [6, 7]. In the present cohort, a higher number of infection events occurred in the BioIntegral group compared with the Omniflow group. However, this observation should be interpreted with caution, as all BioIntegral procedures were performed in infected fields, whereas four patients in the Omniflow group underwent reconstruction in noninfected settings, for example, due to active osteitis outside of the direct operative field. None of these initially noninfected patients developed graft infection during follow‐up. To assess whether this baseline difference disproportionately influenced outcomes, additional exploratory sensitivity analyses were performed. In these Kaplan–Meier estimates of infected cases only, no statistically significant differences were observed between the BioIntegral and Omniflow group in any of the assessed long‐term outcomes.

Notably, in the BioIntegral group, seven patients died after 12 months, and late graft infection (up to 28 months) preceded five of these. This finding underscores the importance of prolonged clinical and imaging surveillance following aortic reconstruction with biologic grafts irrespective of the initial infection status. Although xenograft conduits are most commonly selected for use in infected or potentially contaminated fields, inclusion of mixed indications reflects pragmatic real‐world clinical practice.

Patency outcomes did not differ significantly between graft types. Most patency loss and occlusive events occurred early with few additional events beyond 12 months. This pattern suggests that progressive late conduit degeneration may not be the primary mechanism of graft failure in either group. Instead, in present study unmeasured factors potentially related to patient frailty, compromised distal outflow or perioperative conditions may play a more prominent role, as has been reported in previous studies [20, 21]. Larger‐scale studies with extended follow‐up are warranted to determine whether this pattern is consistent across different biological graft materials.

Despite frequent loss‐of‐patency events, major amputation rates remained low, suggesting secondary peripheral interventions (thrombolysis, thrombectomy, secondary bypass and patch angioplasty) were often feasible to preserve limb perfusion. In contrast, reinterventions involving the central aortic reconstruction were rare and associated with poor outcomes.

Overall mortality in this cohort was high, with 30‐day mortality of 19% in the BioIntegral group and 30% in the Omniflow group, exceeding previously reported rates of 8% for Omniflow in aortic reconstruction and 13.5% after open abdominal aortic graft infection surgery [11, 22]. Of these early deaths, two of four in the BioIntegral group, and three of seven in the Omniflow group were considered graft related. This most likely reflects severe baseline risk and operative complexity of patients undergoing open aortic reconstruction, many of whom presented with active infection and/or aortoenteric pathology. As a predominantly tertiary referral cohort, this population may have included more severe and technically demanding cases than prior series, with aortoenteric pathology, urgent procedures and substantial comorbidity. Given the limited sample size and low event counts, differences in mortality should not be interpreted as necessarily conduits‐related but rather as reflecting case mix and baseline operative risk.

In our experience, the prefabricated bifurcated BioIntegral graft was generally easy to implant, whereas bifurcated Omniflow reconstructions required construction on the back table using two separate grafts. In addition, the thicker wall of the Omniflow graft made suturing somewhat more demanding. However, we must emphasize that these observations reflect subjective surgical experience rather than true comparative findings of the present study and should therefore be interpreted accordingly. Lastly, BioIntegral was withdrawn from commercial distribution in 2022. The reasons for discontinuation were said to not be related to published safety concerns. Nevertheless, these long‐term results remain clinically relevant for patients who have received these grafts in the past and their treating clinicians. Alternative bovine pericardial materials (e.g., patches) remain available, although extrapolation to other bovine pericardial conduits should be done cautiously.

This study has its limitations. Most importantly, this cohort study has a small sample size with low event counts, which is an inherent consequence of the rarity of open (often infected) aortic reconstruction and the requirement for specifically selected conduit materials. Accordingly, the wide confidence intervals observed in Kaplan–Meier estimates and hazard ratios limit the ability to draw firm comparative conclusions in long‐term analyses particularly for infection‐related outcomes, or perform subgroup analyses. Heterogeneity in indication, urgency, and center‐ and surgeon‐level practice could have led to residual confounding that we were not able to correct for in multivariate analyses due to the low event counts. Second, this present cohort represents patients selected for reconstruction with BioIntegral or Omniflow xenografts only and does not reflect the overall treatment algorithm or the relative frequency of alternative reconstruction techniques across all participating centers. Lastly, this study combined a retrospectively and prospectively collected cohort. Although both cohorts originated from Dutch hospitals with similar practices and protocols, residual bias related to changes in clinical practice, perioperative management, complication mitigation and surgeon‐ and center‐specific management cannot be excluded. Therefore, results should be viewed as descriptive and hypothesis‐generating.

This study has several strengths. It provides clinically relevant data for a rare, high‐risk surgery indication. For this reason, data were analyzed and portrayed to provide value in future meta‐analyses on this subject (Supporting Information S1: Table I). Standardized follow‐up with imaging and reporting of clinically meaningful endpoints further enhances interpretability to clinical practice. Lastly, reporting was performed according to STROBE‐ and ESVS/SVS‐guidelines [3, 15].

## 5 Conclusion

In this multicenter cohort study, BioIntegral bovine pericardial grafts and Omniflow II conduits showed similar outcomes for in situ aortic reconstruction with no statistically significant difference in patency, mortality, or freedom from major amputation. Given the limited sample size and event counts, it should not be interpreted as equivalent performance. These findings should be interpreted as descriptive and hypothesis‐generating in a field where evidence remains scarce.

## Author Contributions

**Tijmen W. Kraai:** conceptualization, investigation, writing – original draft, methodology, visualization, formal analysis, data curation, validation, project administration. **Eline Reinders Folmer I:** conceptualization, investigation, writing – review and editing, validation, formal analysis, data curation, project administration. **Barzi Gareb:** methodology, writing – review and editing, conceptualization, investigation, formal analysis, software, supervision. **David J. Liesker:** data curation, conceptualization, writing – review and editing, validation, formal analysis, investigation. **Ben R. Saleem:** conceptualization, investigation, writing – review and editing, funding acquisition, validation, formal analysis, project administration, supervision. **Clark J. Zeebregts:** funding acquisition, conceptualization, investigation, writing – review and editing, validation, formal analysis, project administration, supervision.

## Funding

The authors have nothing to report.

## Conflicts of Interest

The authors declare no conflicts of interest.

## Supporting information


Supporting Information S1

Supporting Information S2

## Data Availability

The data that support the findings of this study are available on request from the corresponding author. The data are not publicly available due to privacy or ethical restrictions.
