# Supplementary material for: Long‐Term Outcomes of Omniflow II and BioIntegral Xenografts for Open Aortic Reconstruction in Predominantly Infected Settings: A Multicenter Cohort Study
Source: World J Surg. 2026 Sep 12;50(10):3129–37. doi: 10.1002/wjs.70559 (PMC13662509; doi:10.1002/wjs.70559)
Supplement: Supplementary file 1 [file WJS-50-3129-s001.docx]

| Material | INAA or VGEI | Blowout* | Location  infection | Previous material | Cultures  preoperative | Cultures  Peroperative | Bacteria from cultures | Leukocytes preop | CRP preop | AB  preop |
| --- | --- | --- | --- | --- | --- | --- | --- | --- | --- | --- |
| Omniflow | VGEI | no | Femoral left | Bovine | Positive |  | S. aureus,  morganella,  enterobacter cloacae complex,  E. coli | 6,6 |  | Yes |
| Omniflow | VGEI | no | Central | Dacron | Positive | Positive | c. albicans, e. faecium, c. tropicalis (uitgehaalde prothese) | 6,2 | 158 | Yes |
| Omniflow | VGEI | no | Ileofemoral  right | Dacron | Positive | Positive | s. epiderdimis | 13,5 | 84 | Yes |
| Omniflow | VGEI | no | Central | EVAR | None | Positive | e. faecium, s. hominis, m. luteus, candida glabrata | 13,6 | 91 | Yes |
| Omniflow | INAA | no | Central | None | None | None |  | 16,5 | 2,4 | No |
| Omniflow | non-infected | no | N/A | None | None |  |  | 9 |  | No |
| Omniflow | non-infected | no | N/A | None | None |  |  | 7,3 | 53 | No |
| Omniflow | VGEI | no | Central | Dacron | Positive |  | e. faecium, | 15,5 | 149 | Yes |
| Omniflow | VGEI | no | Ileofemoral right | EVAR | None | Positive | e. faecalis,  m. luteus | 11,9 | 101 | Yes |
| Omniflow | VGEI | no | Central | Dacron | Negative | Negative |  | 7,8 |  | No |
| Omniflow | INAA | yes | Central | None | Negative | Negative |  | 12,4 | 136 | No |
| Omniflow | VGEI | no | Central | Silvercoated Dacron | Positive | Negative | staph. hominis | 7,9 | 10 | Yes |
| Omniflow | INAA | no | Central | None | Positive | Negative | staph. aureus | 6,3 | 79 | Yes |
| Omniflow | VGEI | no | Central | EVAR | Negative | Positive | m. bovis / tbc (later bekend). | 8,6 | 128 | Yes |
| Omniflow | VGEI | yes | femoropopliteal | Dacron | None | Positive | e.coli, e. faecium, p. distasonis,  b. fragilis | 7,3 |  | No |
| Omniflow | INAA | yes | Central | None | Positive | Negative | Staph. aureus | 14,6 | 224 | Yes |
| Omniflow | non-infected | no | N/A | None | None | Negative |  | 11,3 | 2 | No |
| Omniflow | VGEI | yes | Central | Dacron | Positive | Positive | s. vestibularis,  s. sanguinis | 11,2 | 1 | No |
| Omniflow | VGEI | no | Central | EVAR | Negative | Negative |  | 6,6 |  | No |
| Omniflow | non-infected | yes | N/A | None | None | None |  | 10,8 | 32 | No |
| Omniflow | INAA | no | Central | None | None | Negative |  | 8 | 41 | Yes |
| Omniflow | VGEI | no | Central | EVAR | None | Positive | klebsiella |  |  | Yes |
| Omniflow | VGEI | yes | Central | EVAR | None | Negative |  |  |  | Yes |
| BioIntegral | VGEI | no | Central | EVAR | Positive | Positive | Enterococcus faecium, lactobacullus rhamnosus, candida albicans,  Serratia marcesens | 4,9 | 50 | Yes |
| BioIntegral | VGEI | no | Central | Dacron | Positive | Positive | etercoccus faecium,  candida albicans,  Str. anginosus,  actinomyces odontolyticus | 17,8 | 36 | Yes |
| BioIntegral | VGEI | no | Central | EVAR | Positive | Positive | e. coli,  staph. Lugdinensis, enterococcus faecalis, prevotella denticolam atopbium parvulum | 9 | 221 | Yes |
| BioIntegral | VGEI | no | Central | Dacron | Positive | Positive | Proteus mirabilis, enterococcus faecalis, str. anginosus, prevotella denticola, candida glabrata | 14,6 | 100 | Yes |
| BioIntegral | VGEI | yes | Central | EVAR | None | Positive | Staphylococcus epidermis | 5,4 | 138 | Yes |
| BioIntegral | VGEI | no | Central | Dacron | Positive | Negative | Cornyebacterium amycolatum, s.simulans | 9,9 | 5 | Yes |
| BioIntegral | VGEI |  |  | Dacron | Positive |  | Combination | 7,6 | 220 |  |
| BioIntegral | mAAA |  |  | None | Positive |  | Combination | 18,1 | 170 |  |
| BioIntegral | VGEI | yes | Central | Dacron | Negative | Positive | Streptococcus anginosus, campylobacter gracilis | 21,6 | 193 | Yes |
| BioIntegral | VGEI | yes | Femorofemoral crossover | Dacron | Positive | Positive | staphylococcis hominis | 6,6 | 17 | Yes |
| BioIntegral | VGEI | no | Central | Dacron | Negative | Negative |  | 11,6 | 20 | Yes |
| BioIntegral | mAAA | no | Central | None | None | None |  | 8 | 138 | Yes |
| BioIntegral | VGEI | no | Central | EVAR | None | Positive | Listeria | 11,3 | 23 | No |
| BioIntegral | mAAA | no | Central | None | Negative | Positive | Propionibactericum acnes | 16 | 24 | Yes |
| BioIntegral | VGEI | no | Central | Dacron | Negative | Positive | Apergillus | 10,4 | 16 | Yes |
| BioIntegral | VGEI | no | Central | Dacron | Positive | Positive | Aerobe, gram positive combination | 13,5 | 119 | Yes |
| BioIntegral | VGEI | no | Central | EVAR | Positive | Positive | Staphylococcus epidermis | 12,1 | 297 | Yes |
| BioIntegral | VGEI | yes | Central | Silvercoated Dacron | Positive | Positive | Staphylococcus aureus, cornyebacterium amycolatum | 10,8 | 34 | Yes |
| BioIntegral | VGEI | yes | Central | Dacron | Positive | Positive | Propionibactericum acnes | 10,1 | 18 | Yes |
| BioIntegral | VGEI | no | Central | EVAR | Positive | Positive | Staph. Epidermis, candida albicans | 9,1 | 43 | Yes |
| BioIntegral | VGEI | yes | Central and femorofemoral crossover | Dacron | Positive | Positive | cornyebacterium striatum, helcoccus kunzii, bacteriudes fragilis | 12,2 | 166 | No |

INAA = Infective Native Aortic Aneurysm, VGEI = Vascular Graft and/or Endograft Infection

*Blowout or active bleeding at presentation
