# Supplementary material for: Long‐Term Outcomes of Omniflow II and BioIntegral Xenografts for Open Aortic Reconstruction in Predominantly Infected Settings: A Multicenter Cohort Study
Source: World J Surg. 2026 Sep 12;50(10):3129–37. doi: 10.1002/wjs.70559 (PMC13662509; doi:10.1002/wjs.70559)
Supplement: Supplementary file 2 [file WJS-50-3129-s002.docx]

**Primary patency**

| Material | Time (months) | n at risk | n event | n censor | Survival (%) | Lower bound 95CI (%) | Upper bound 95CI (%) |
| --- | --- | --- | --- | --- | --- | --- | --- |
| Overall | 12 | 23 | 10 | 11 | 74.6 | 62.0 | 89.8 |
| Overall | 24 | 15 | 2 | 6 | 67.3 | 53.3 | 85.0 |
| Overall | 60 | 7 | 2 | 8 | 53.6 | 35.6 | 80.7 |
| BioIntegral | 12 | 13 | 3 | 5 | 85.4 | 71.5 | 100 |
| BioIntegral | 24 | 8 | 1 | 4 | 77.7 | 60.0 | 100 |
| BioIntegral | 60 | 4 | 2 | 4 | 49.9 | 25.0 | 99.7 |
| Omniflow | 12 | 10 | 7 | 6 | 62.4 | 43.4 | 89.8 |
| Omniflow | 24 | 7 | 1 | 2 | 55.5 | 36.1 | 85.4 |
| Omniflow | 60 | 3 | 0 | 4 | 55.5 | 36.1 | 85.4 |

**Primary assisted patency**

| Material | Time (months) | n at risk | n event | n censor | Survival (%) | Lower bound 95CI (%) | Upper bound 95CI (%) |
| --- | --- | --- | --- | --- | --- | --- | --- |
| Overall | 12 | 22 | 8 | 14 | 79.6 | 67.7 | 93.6 |
| Overall | 24 | 15 | 1 | 6 | 75.2 | 61.7 | 91.5 |
| Overall | 60 | 7 | 0 | 8 | 75.2 | 61.7 | 91.5 |
| BioIntegral | 12 | 12 | 2 | 7 | 90.5 | 78.8 | 100 |
| BioIntegral | 24 | 7 | 1 | 4 | 80.4 | 61.4 | 100 |
| BioIntegral | 60 | 3 | 0 | 4 | 80.4 | 61.4 | 100 |
| Omniflow | 12 | 10 | 6 | 7 | 68.1 | 49.4 | 93.9 |
| Omniflow | 24 | 8 | 0 | 2 | 68.1 | 49.4 | 93.9 |
| Omniflow | 60 | 4 | 0 | 4 | 68.1 | 49.4 | 93.9 |

**Secondary patency**

| Material | Time (months) | n at risk | n event | n censor | Survival (%) | Lower bound 95CI (%) | Upper bound 95CI (%) |
| --- | --- | --- | --- | --- | --- | --- | --- |
| Overall | 12 | 24 | 6 | 14 | 83.9 | 72.6 | 96.9 |
| Overall | 24 | 17 | 1 | 6 | 79.7 | 66.8 | 95.0 |
| Overall | 60 | 8 | 0 | 10 | 79.7 | 66.8 | 95.0 |
| BioIntegral | 12 | 13 | 1 | 7 | 95.2 | 86.6 | 100 |
| BioIntegral | 24 | 8 | 1 | 4 | 85.7 | 68.3 | 100 |
| BioIntegral | 60 | 4 | 0 | 5 | 85.7 | 68.3 | 100 |
| Omniflow | 12 | 11 | 5 | 7 | 72.4 | 54.1 | 97.1 |
| Omniflow | 24 | 9 | 0 | 2 | 72.4 | 54.1 | 97.1 |
| Omniflow | 60 | 4 | 0 | 5 | 72.4 | 54.1 | 97.1 |

**Amputation**

| Material | Time (months) | n at risk | n event | n censor | Survival (%) | Lower bound 95CI (%) | Upper bound 95CI (%) |
| --- | --- | --- | --- | --- | --- | --- | --- |
| Overall | 12 | 27 | 3 | 14 | 92.6 | 84.9 | 100 |
| Overall | 24 | 18 | 2 | 7 | 85.4 | 74.0 | 98.5 |
| Overall | 60 | 9 | 0 | 10 | 85.4 | 74.0 | 98.5 |
| BioIntegral | 12 | 13 | 2 | 6 | 89.9 | 77.6 | 100 |
| BioIntegral | 24 | 8 | 1 | 4 | 81.8 | 64.5 | 100 |
| BioIntegral | 60 | 4 | 0 | 5 | 81.8 | 64.5 | 100 |
| Omniflow | 12 | 14 | 1 | 8 | 95.7 | 87.7 | 100 |
| Omniflow | 24 | 10 | 1 | 3 | 88.8 | 75.0 | 100 |
| Omniflow | 60 | 5 | 0 | 5 | 88.8 | 75.0 | 100 |

**Mortality**

| Material | Time (months) | n at risk | n event | n censor | Survival (%) | Lower bound 95CI (%) | Upper bound 95CI (%) |
| --- | --- | --- | --- | --- | --- | --- | --- |
| Overall | 12 | 27 | 16 | 1 | 63.6 | 50.9 | 79.6 |
| Overall | 24 | 20 | 3 | 4 | 56.1 | 43.0 | 73.1 |
| Overall | 60 | 9 | 6 | 6 | 36.1 | 23.1 | 56.4 |
| BioIntegral | 12 | 13 | 7 | 1 | 66.7 | 49.23 | 90.2 |
| BioIntegral | 24 | 9 | 3 | 1 | 50.0 | 32.0 | 78.0 |
| BioIntegral | 60 | 4 | 4 | 2 | 25.0 | 11.0 | 57.0 |
| Omniflow | 12 | 14 | 9 | 0 | 60.9 | 43.9 | 84.5 |
| Omniflow | 24 | 11 | 0 | 3 | 60.9 | 43.9 | 84.5 |
| Omniflow | 60 | 5 | 2 | 4 | 47.3 | 29.3 | 76.4 |

**Infection**

| Material | Time (months) | | n at risk | n event | n censor | Survival (%) | Lower bound 95CI (%) | Upper bound 95CI (%) |
| --- | --- | --- | --- | --- | --- | --- | --- | --- |
| Overall | | 12 | 26 | 4 | 14 | 87.6 | 76.9 | 99.8 |
| Overall | | 24 | 16 | 4 | 6 | 72.0 | 57.0 | 90.9 |
| Overall | | 60 | 8 | 2 | 7 | 62.7 | 46.3 | 84.8 |
| BioIntegral | | 12 | 13 | 2 | 6 | 88.2 | 74.2 | 100 |
| BioIntegral | | 24 | 7 | 3 | 3 | 63.7 | 42.1 | 96.3 |
| BioIntegral | | 60 | 4 | 2 | 2 | 43.7 | 22.3 | 85.7 |
| Omniflow | | 12 | 13 | 2 | 8 | 87.1 | 71.8 | 100 |
| Omniflow | | 24 | 9 | 1 | 3 | 79.1 | 60.5 | 100 |
| Omniflow | | 60 | 4 | 0 | 5 | 79.1 | 60.5 | 100 |
